# Preference for enzalutamide capsules versus tablet pills in patients with prostate cancer

**DOI:** 10.1111/iju.14101

**Published:** 2019-09-18

**Authors:** Sahoko Ninomiya, Takashi Kawahara, Tomoyuki Tatenuma, Yasuhide Miyoshi, Hiroshi Miyamoto, Masahiro Yao, Hiroji Uemura

**Affiliations:** ^1^ Departments of Urology and Renal Transplantation Yokohama City University Medical Center Yokohama Kanagawa Japan; ^2^ Department of Urology Yokohama City University Graduate School of Medicine Yokohama Kanagawa Japan; ^3^ Departments of Pathology and Laboratory Medicine, Urology, and Oncology University of Rochester Medical Center Rochester New York USA

**Abbreviations & Acronyms**
CRPC: castration‐resistant prostate cancer
ENZ: enzalutamide
PFS: progression‐free survival
PRO: patient‐reported outcome

ENZ has been widely used for the treatment of metastatic CRPC since the prolongation of PFS or overall survival was shown in the PREVEIL and AFFIRM studies.^1^, ^2^ The PROSPER trial also confirmed prolonged radiographic PFS in patients with non‐metastatic CRPC.^3^ Because of its low toxicity, ENZ has also been often prescribed to elderly patients who are, for example, unfit for chemotherapy.^1^, ^2^, ^3^ In contrast, ENZ treatment has been shown to be associated with adverse events, including fatigue and decreased appetite, that might significantly reduce their quality of life in approximately 30–50% of patients in these studies.^2^, ^3^ Indeed, dose reduction is occasionally required for some patients.^4^ Thus, controlling these symptoms, while maintaining therapeutic effects without dose reduction, is important for allowing patients to benefit from ENZ treatment.

ENZ has been approved for the treatment of CRPC in 91 countries. In June 2018, the form of ENZ was changed from a 40‐mg capsule (21 × 10 mm in size) to a 40‐mg tablet pill (10.1 mm in diameter) or an 80‐mg tablet pill (17.2 × 9.1 mm) in Japan and Germany. Our recent case study showed that changing the form of ENZ for a patient not only improved medication adherence, but also alleviated side‐effects, such as fatigue, achieving full‐dose, on‐schedule administration of ENZ.^5^ The present study examined the differences between these two forms of medication, using PROs. The institutional review board of Yokohama City University Medical Center (Yokohama, Japan) approved this study (No. B181100015).

In a total of 14 healthy men who were asked to take placebos of the same sizes as the ENZ capsule (40 mg) and tablet (40 mg), we first assessed the ease of taking them by comparing the volume of water required to swallow and their preference. The placebo capsules and multivitamin supplement tablets used were commercially available non‐prescription drugs (Table S1). The water amount was significantly reduced from 59.2 mL (four capsules) to 33.8 mL (four tablets; *P* < 0.001; Fig. S1), and 13 (92.9%) of the 14 men preferred tablets to capsules.

To further assess the potential benefits of changing its form, a total of 25 CRPC patients to whom both ENZ capsules and tablets were given at Yokohama City University Medical Center (Yokohama, Japan) were enrolled in the present study. The median (mean ± standard deviation) age was 75 years (76.5 ± 7.3 years). These patients were then asked to complete the questionnaire, along with the Cancer Fatigue Scale (i.e. physical/affective/cognitive subscales and total scale score) developed by Okuyama *et al*.^6^ All patients completed the questionnaire 1 month after ENZ capsule or tablet intake (Fig. S2). Overall, 21 (84.0%) patients preferred tablets to capsules, whereas both forms were equally acceptable in the remaining three (12.0%) patients (Fig. 1a). For other factors examined, the patients either preferred tablets or had no preference between capsules and tablets. The affective and total scores were significantly lower for tablets than for capsules, whereas there were no significant differences in the physical and cognitive scores between the two forms (Fig. 1b). Two questions showed significant differences between capsules and tablets (Table S2). For the 25 CRPC patients, the mean number of drugs administered was six, with a total of 11 capsules and/or tablet pills. The detailed mechanisms underlying why the tablet drug forms showed a favorable score are unclear, although the rapid increase in the plasma drug concentration as a result of capsule ingestion might be involved.

**Figure 1:**
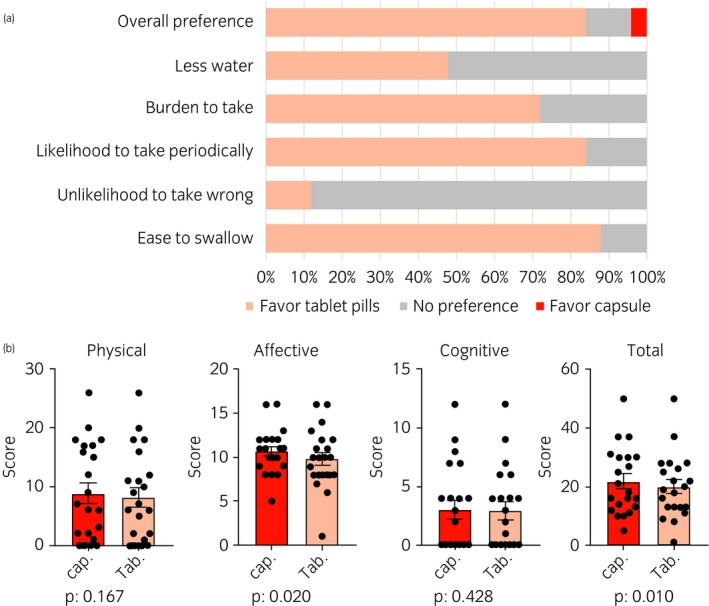
(a) Preference between capsule and tablet. (b) Cancer fatigue scale between capsule and tablet.

The change in the form of ENZ was thus found to result in a reduction of the rate of not only the difficulty associated with taking the drug, but also fatigue. Because ENZ has been changed to a tablet form in Japan, with the capsule form not available, a reverse study cannot be carried out. Despite this limitation, in comparison with the capsules, less water was required when taking the tablets.

## Conflict of interest

None declared.

## Supporting information


**Figure S1.** Water intake between capsule and tablet.

**Figure S2.** Summary of this study.

**Table S1.** Placebo capsules and same‐size drug forms were used as commercially available drugs.

**Table S2.** Questions that showed significant differences between capsule and tablet forms.
