# Supplementary figures and images for: Preference for enzalutamide capsules versus tablet pills in patients with prostate cancer
Source: Int J Urol. 2019 Sep 18;26(12):1161–2. doi: 10.1111/iju.14101 (PMC6916586; doi:10.1111/iju.14101)

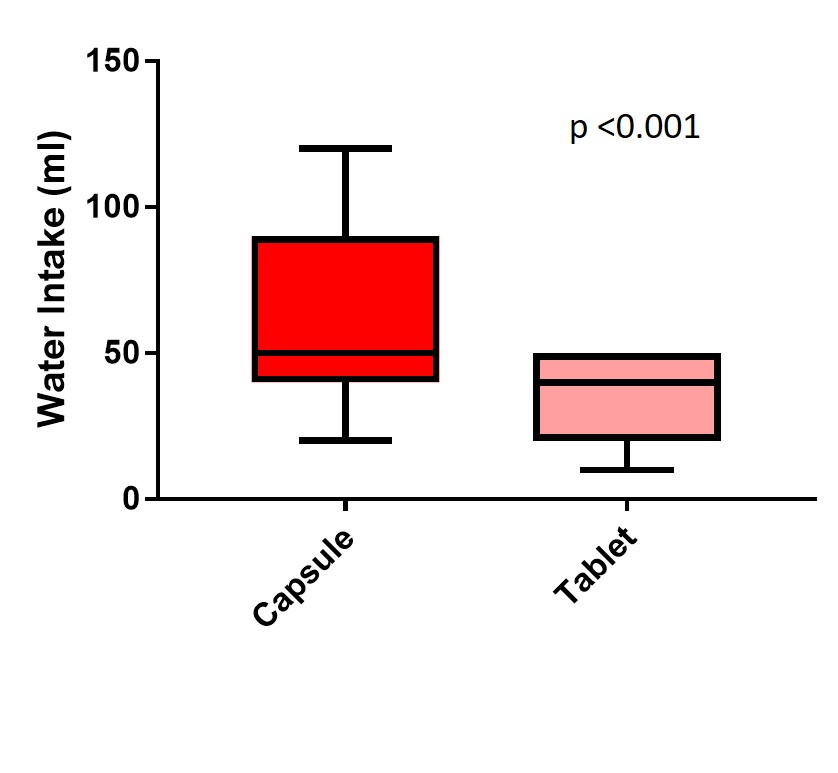

Supplement: Supplementary file 1 — Figure S1. Water intake between capsule and tablet. [file IJU-26-1161-s001.jpg]
