# Supplementary material for: Preference for enzalutamide capsules versus tablet pills in patients with prostate cancer
Source: Int J Urol. 2019 Sep 18;26(12):1161–2. doi: 10.1111/iju.14101 (PMC6916586; doi:10.1111/iju.14101)
Supplement: Supplementary file 2 — Figure S2. Summary of this study. [file IJU-26-1161-s002.pptx]

## Slide 1
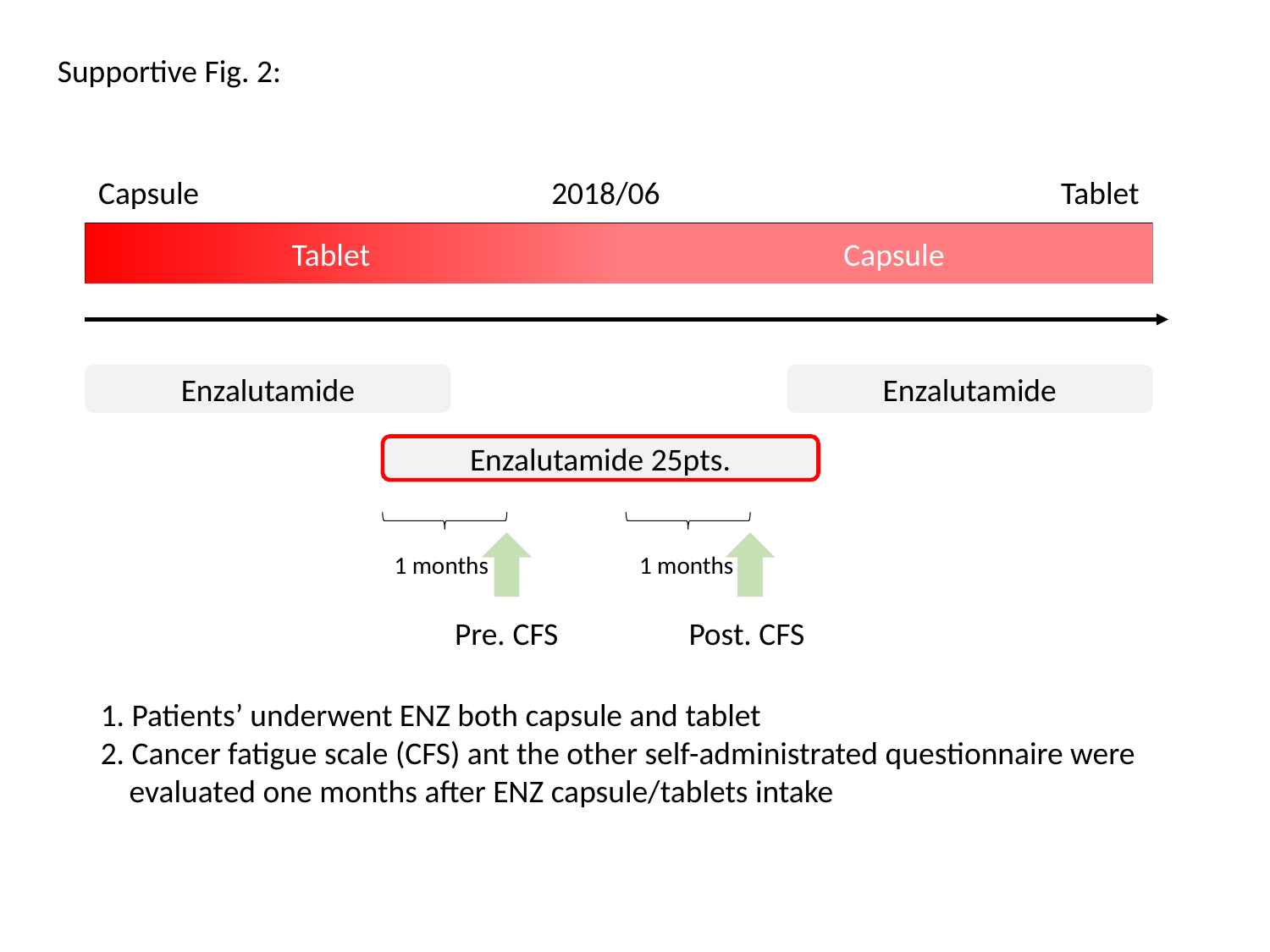

Supportive Fig. 2:
Capsule
2018/06
Tablet
Tablet Capsule
Enzalutamide
Enzalutamide
Enzalutamide 25pts.
1 months
1 months
Pre. CFS
Post. CFS
 1. Patients’ underwent ENZ both capsule and tablet
 2. Cancer fatigue scale (CFS) ant the other self-administrated questionnaire were
 evaluated one months after ENZ capsule/tablets intake
