# Supplementary material for: Preference for enzalutamide capsules versus tablet pills in patients with prostate cancer
Source: Int J Urol. 2019 Sep 18;26(12):1161–2. doi: 10.1111/iju.14101 (PMC6916586; doi:10.1111/iju.14101)
Supplement: Supplementary file 3 — Table S1. Placebo capsules and same‐size drug forms were used as commercially available drugs. [file IJU-26-1161-s003.pdf]

Supportive table 1:

Capsule: #0 capsule (NISSIN MEDICAL INSTRUMENT CO., Osaka, Japan)
Tablet: Dear-Natura Vitamin C, E, A (Asahi Group Foods, Tokyo, Japan)
