# Supplementary material for: Preference for enzalutamide capsules versus tablet pills in patients with prostate cancer
Source: Int J Urol. 2019 Sep 18;26(12):1161–2. doi: 10.1111/iju.14101 (PMC6916586; doi:10.1111/iju.14101)
Supplement: Supplementary file 4 — Table S2. Questions that showed significant differences between capsule and tablet forms. [file IJU-26-1161-s004.pdf]

Supportive table 2:

Original Questionnaire
3. Do you feel exhausted?
11. Can you concentrate on certain things?

Japanese translated questionnaire
3. ぐったりと感じますか？
11. 物事に集中することはできますか？
